# Supplementary material for: Neural parameter calibration and uncertainty quantification for epidemic forecasting
Source: PLoS One. 2024 Oct 17;19(10):e0306704. doi: 10.1371/journal.pone.0306704 (PMC12007818; doi:10.1371/journal.pone.0306704)
Supplement: S1 Appendix — (PDF) [file pone.0306704.s001.pdf]

Supporting Information

Methodology

Initialising the neural network

We can initialise the neural network to a particular distribution on its output by drawing random variables $\boldsymbol{\Lambda}_0$ from the target distribution and training the neural network to output the target value before the run. This can be done using the simple loss function

$$J = \|\hat{\boldsymbol{\Lambda}} - \boldsymbol{\Lambda}_0\|_2, \tag{14}$$

which typically only takes a few seconds. When running the neural network multiple times from different initialisations the resulting distribution of initial values will approach the target distribution as the number of chains goes to infinity. In Fig S1 we show the initial values for the SIR parameters (β, τ, α) for 100 different initialisations; as is visible, the distributions are approximately uniform on the intervals as specified.

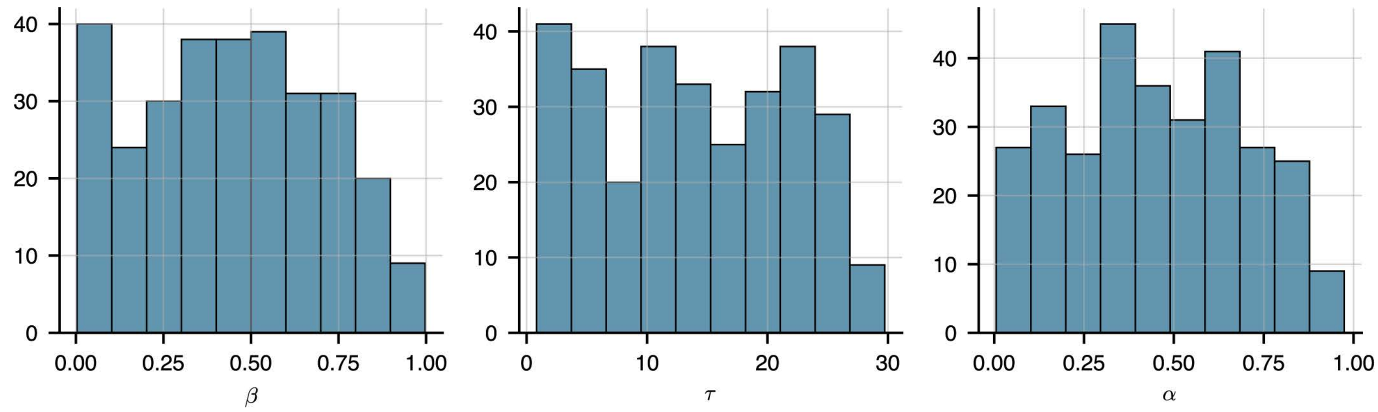

Fig S1. Initial value distribution on β, τ, and α for the neural network. Each parameter is initialised to a uniform distribution.

Running the MCMC

We initialise the MCMC with the same initial distribution as the neural network. For the SIR experiments, we run 50 randomly initialised chains for 10.000 steps each. Fig S2 shows the Gelman-Rubin statistic over time for the 3-dimensional SIR experiment. Note that the value for α does not converge to below 1.2, which seems to indicate the MCMC sampler getting caught in local minima, preventing complete mixing.

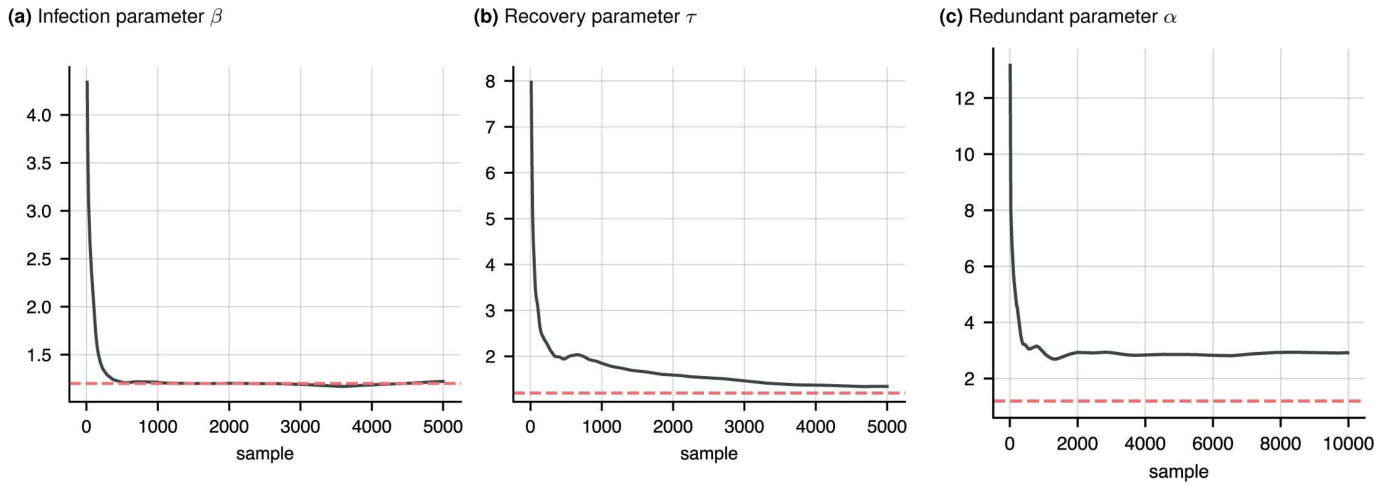

Fig S2. Gelman-Rubin statistics for the MCMC chains for the parameters (β, τ, α). The red dotted line indicates a value of 1.2.

Modelling the spread of COVID-19 in Berlin

The ODE model used to model the spread of COVID-19 in Berlin was previously presented in [13], and is visualised in figure 2 in the main manuscript:

$$
\begin{aligned}
\frac{\mathrm{dS}}{\mathrm{d}t} &= -\lambda_{\mathrm{E}}\mathrm{SI} + \lambda_{\mathrm{S}}\mathrm{Q_S} - \lambda_{\mathrm{Q}}\mathrm{S} & \frac{\mathrm{dD}}{\mathrm{d}t} &= \lambda_{\mathrm{D}}\mathrm{C} \\
\frac{\mathrm{dE}}{\mathrm{d}t} &= \lambda_{\mathrm{E}}\mathrm{SI} - \lambda_{\mathrm{E}}\mathrm{I} - \lambda_{\mathrm{Q}}\mathrm{E} & \frac{\mathrm{dQ_E}}{\mathrm{d}t} &= -\lambda_{\mathrm{I}}\mathrm{Q_E} + \lambda_{\mathrm{Q}}\mathrm{E} \\
\frac{\mathrm{dI}}{\mathrm{d}t} &= \lambda_{\mathrm{I}}\mathrm{E} - \lambda_{\mathrm{R}}\mathrm{I} - \lambda_{\mathrm{SY}}\mathrm{I} - \lambda_{\mathrm{Q}}\mathrm{I} & \frac{\mathrm{dQ_I}}{\mathrm{d}t} &= \lambda_{\mathrm{I}}\mathrm{Q_E} + \lambda_{\mathrm{Q}}\mathrm{I} - \lambda_{\mathrm{SY}}\mathrm{Q_I} - \lambda_{\mathrm{R}}\mathrm{Q_I} \\
\frac{\mathrm{dR}}{\mathrm{d}t} &= \lambda_{\mathrm{R}}\left(\mathrm{I} + \mathrm{SY} + \mathrm{H} + \mathrm{C} + \mathrm{Q_I}\right) & \frac{\mathrm{dCT}}{\mathrm{d}t} &= \lambda_{\mathrm{SY}}\mathrm{I} + (\lambda_{\mathrm{CT}} - \lambda_{\mathrm{Q}})(\mathrm{S} + \mathrm{E} + \mathrm{I}) \\
\frac{\mathrm{dSY}}{\mathrm{d}t} &= \lambda_{\mathrm{SY}}\left(\mathrm{Q_I} + \mathrm{I}\right) - \lambda_{\mathrm{R}}\mathrm{SY} - \lambda_{\mathrm{H}}\mathrm{SY} & \frac{\mathrm{dQ_S}}{\mathrm{d}t} &= -\lambda_{\mathrm{S}}\mathrm{Q_S} + \lambda_{\mathrm{Q}}\mathrm{S} \\
\frac{\mathrm{dH}}{\mathrm{d}t} &= \lambda_{\mathrm{H}}\mathrm{SY} - \lambda_{\mathrm{R}}\mathrm{H} - \lambda_{\mathrm{C}}\mathrm{H} & \frac{\mathrm{d}\lambda_{\mathrm{Q}}}{\mathrm{d}t} &= \lambda_{\mathrm{q}}\lambda_{\mathrm{CT}}\mathrm{CT} \\
\frac{\mathrm{dC}}{\mathrm{d}t} & \lambda_{\mathrm{C}}\mathrm{H} - \lambda_{\mathrm{R}}\mathrm{C} - \lambda_{\mathrm{D}}\mathrm{C}
\end{aligned}
\tag{15}
$$

Here, each compartment $i = \mathrm{S}, ..., \mathrm{CT}$ represents the density of agents in that compartment, i.e. $\mathrm{S} = \mathrm{s}/N$, where s is the number of agents in that compartment, and N is the total population of Berlin (ca. 3.6×10^6). λ_{q} is the rate of people going into quarantine after being called by the contact tracing agency, and is assumed to be independent of counter-measures. Following [13], we set $\lambda_{\mathrm{q}} = 10.25$. The ABM data does not contain a deceased compartment, as the Robert-Koch Institute changed its classification of ‘deceased’ in mid-2020, in order to better reflect that not all patients diagnosed with COVID-19 at the time of their death had actually died *of* the disease. The mortality rates for this period are unreliable, and are excluded from the model. A

good approximation (leading to similar results) would be to set D = C/3. Also not given are the number of contact-traced agents (CT), and furthermore the ABM data does not distinguish between the three different quarantined compartments. The loss function used to train the neural network thus becomes

$$J = \sum_{t=1}^{L} \big[\alpha_{\mathrm{S}}(\hat{\mathrm{S}} - \mathrm{S})^2 + \alpha_{\mathrm{E}}(\hat{\mathrm{E}} - \mathrm{E})^2 + \alpha_{\mathrm{I}}(\hat{\mathrm{I}} - \mathrm{I})^2 + \alpha_{\mathrm{R}}(\hat{\mathrm{R}} - \mathrm{R})^2 + \alpha_{\mathrm{SY}}(\hat{\mathrm{SY}} - \mathrm{SY})^2$$
$$+ \alpha_{\mathrm{H}}(\hat{\mathrm{H}} - \mathrm{H})^2 + \alpha_{\mathrm{C}}(\hat{\mathrm{C}} - \mathrm{C})^2 + \alpha_{\mathrm{Q}}((\hat{\mathrm{Q}}_{\mathrm{I}} + \hat{\mathrm{Q}}_{\mathrm{S}} + \hat{\mathrm{Q}}_{\mathrm{E}}) - \mathrm{Q})^2\big],$$

with the coefficients α_i given in Eq (12). In Fig S3 we show the calibrations and projections for the S, E, I, R, and Q compartments, which were not shown in the main manuscript.

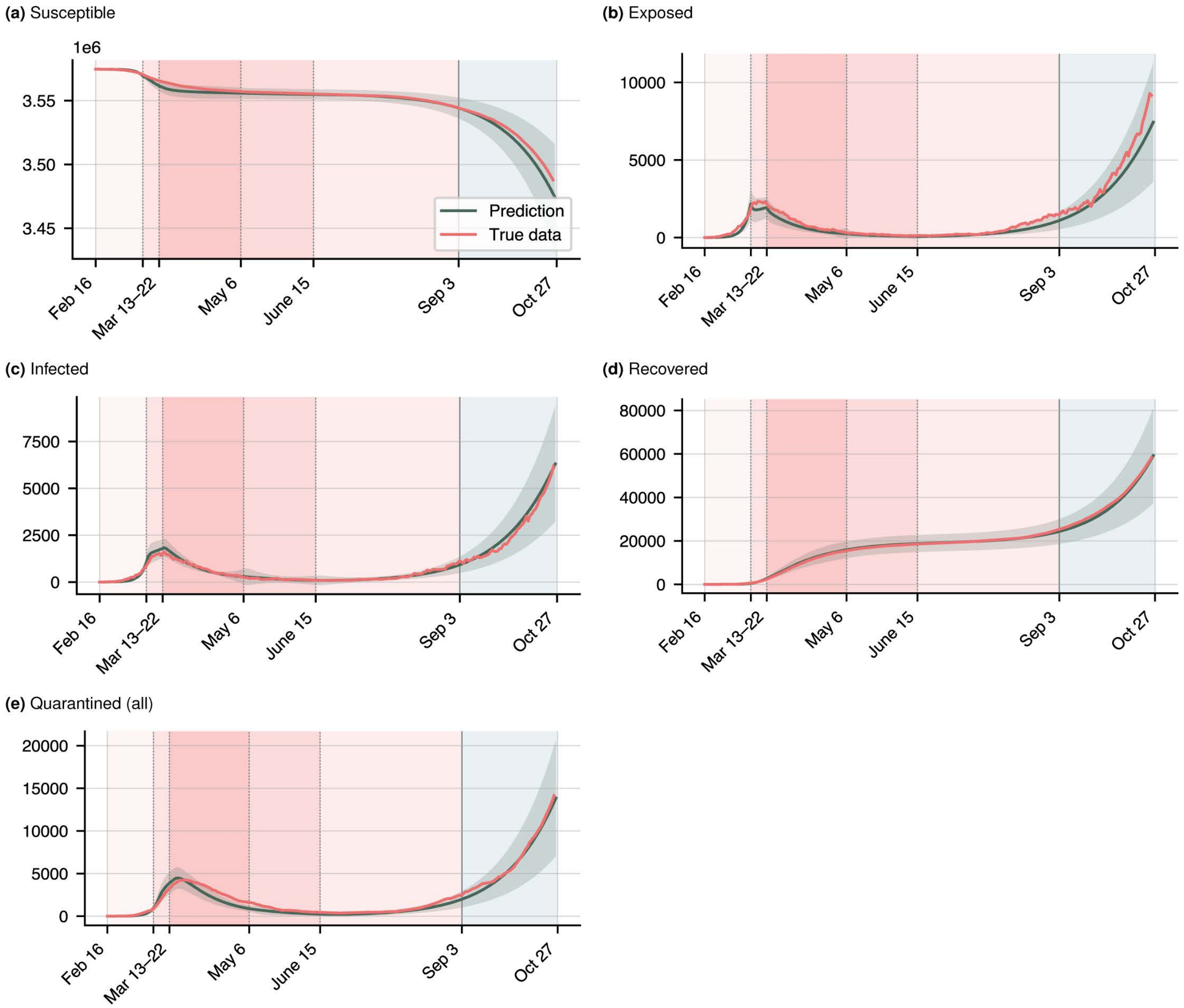

Fig S3. Calibration and projection results pertaining to Tab 2 and Fig 5; shown are the results for the S, E, I, R, and Q compartments. The Q compartment is given by the sum of the Q_S, Q_E, and Q_I compartments.

In figure S4 we show the Gelman-Rubin statistic for the Langevin sampler. For most of the parameters, the chains remain far from the desired value of below 1.2, which would broadly indicate convergence of the chains. Instead, most of the values remain stubbornly high. This again is likely due to the highly non-convex structure of the parameter space, with many different local minima trapping the sampler and preventing mixing (cf. Fig 4).

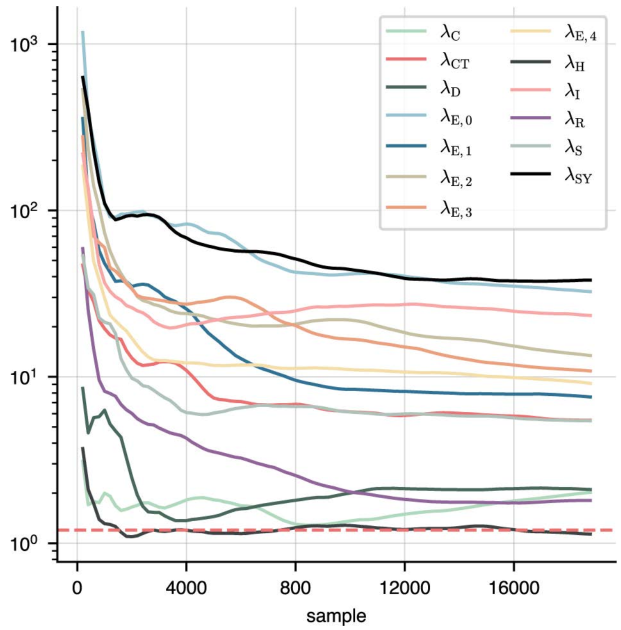

Fig S4. Gelman-Rubin statistics for the MCMC scheme for each of the parameters used to calibrate the ODE model Eq (15). The red dotted value indicates a value of 1.2.
