# Supplementary material for: Neural parameter calibration and uncertainty quantification for epidemic forecasting
Source: PLoS One. 2024 Oct 17;19(10):e0306704. doi: 10.1371/journal.pone.0306704 (PMC12007818; doi:10.1371/journal.pone.0306704)
Supplement: Attachment — Submitted filename: Response_to_reviewers.pdf [file pone.0306704.s002.pdf]

Neural parameter calibration for epidemic forecasing

Response to reviewers

June 20th 2024

Dear Dr Arunachalam,

We thank you and the reviewers for their comments on our submission, which we found most helpful and have incorporated into the manuscript. Please find attached a point-by-point response; changes to the original manuscript are highlighted in orange. We look forward to hearing from you.

Kind regards,

Thomas Gaskin, Tim Conrad, Grigorios A. Pavliotis, Christof Schütte

Reviewer 1 comments

We thank Reviewer 1 for the helpful comments and suggestions.

1. *When the authors mention the importance of uncertainty, new ensemble methods have been proposed, and they seem to improve accuracy in such a context. See Sherratt et al. (2023), among others. A mention of such approaches that are proposed to ensemble predictions of various models should be made in the introduction or the discussion.*

   We have added several references both to ensemble methods and uncertainty quantification in epidemiological modelling using Approximate Bayesian Computation (refs. 26–28, lines 43–47).

2. *The authors state that the method has previously been applied to a diverse set of problems. Specify where and which kind of problems, providing proper citations. It is important to clearly state here that the proposed neural network is not new, which is the novelty here.*

   We have added corresponding citations (14 & 15) and specified the applications (lines 69–71). We have also highlighted the point that the method was developed in previous work in the introduction (lines 28–35), as it is indeed an important one and should be made very clear.

3. *The methodological part is quite technical and I am not sure if the general readers of the journal would be interested in all the details. However, from a statistician's perspective, these technicalities are valuable. I suggest considering placing them in the Appendix.*

   We considered this and attempted to restructure the article to move some of the more technical details into the appendix and pushing the main results to the Berlin dataset to the beginning of the article. However, we quickly found the paper to be difficult to read, since multiple references to the appendix would need to be made, and the flow of the manuscript becoming too non-linear. We have thus decided to keep the technicalities in the first section, as originally submitted.

4. *Page 8, write figures 2 and 4 with capital F. On page 9, write Table 2 with capitals t. Appendix Page 12: In the caption of Figure S3, write Table 2 with capital t.*

   We thank the reviewer for the close reading of the manuscript and attention to detail, and have corrected these points.

Reviewer 2 comments

We also thank Reviewer 2 for the helpful comments and suggestions.

1. *Even though it is an application of the method proposed elsewhere [13][14], it would be good to highlight why it yields a valid posterior distribution, especially 'in the limit of infinitely many chains'?*

   This touches upon many of the things we are in the process of discussing. On the face of it, the answer to this question is simple: our method can be understood as a sophisticated grid search, that scans the parameter space and collects the samples it finds. In the limit of infinitely many chains, it can be expected to at some point hit every parameter (at least in the domain of initialisation) and thus converge to the true likelihood function. However, this argument is lacking technical details and a rigorous proof of convergence. We have added a few sentences to the discussion clarifying that we are working on a more formal convergence analysis of our method (lines 319–321).

2. *When comparing running times, e.g. in Table 1, does time of the neural scheme include the neural network training time?*

   Yes – in fact, the training time is the only relevant time to measure, since evaluating the trained neural network is practically instantaneous. Once the neural network is trained, it is a function that extracts parameters from a dataset – but the training process itself can be leveraged to obtain probability densities on the estimates, thereby also achieving what a sampler would do.

3. *On line 222, it should be 'estimate' instead of 'estimates'.* This has been corrected.

Editor comments

1. *Please state what role the funders took in the study.*

   The funders had no role in study design, data collection and analysis, decision to publish, or preparation of the manuscript. There was no additional external funding received for this study.

2. *We note that the grant information you provided in the 'Funding Information' and 'Financial Disclosure' sections do not match.*

   The following is the correct funding statement:

   > TG was funded by the University of Cambridge School of Physical Sciences VC Award via DAMTP and the Department of Engineering, and supported by EPSRC grants EP/P020720/2 and EP/R018413/2. TC and CS were funded by the Deutsche Forschungsgemeinschaft (DFG) under Germany's Excellence Strategy through grant EXC-2046 The Berlin Mathematics Research Center MATH+ (project no. 390685689) and via the grant MODUS-COVID by the German Ministry for Education and Research (BMBF) (grant number 031L0302C). GP is partially supported by the Frontier Research Advanced Investigator Grant ERC grant Machine-aided general framework for fluctuating dynamic density functional theory.

   We would also like to thank Ms. Wulkow for her *assistance* in obtaining the data – her support was not financial. We have amended the acknowledgements statement accordingly.
